# Reproducibility and discrimination of different indices of insulin sensitivity and insulin secretion

**DOI:** 10.1371/journal.pone.0258476

**Published:** 2021-10-22

**Authors:** Sarah Hudak, Philipp Huber, Apostolia Lamprinou, Louise Fritsche, Norbert Stefan, Andreas Peter, Andreas L. Birkenfeld, Andreas Fritsche, Martin Heni, Robert Wagner

**Affiliations:** 1 Division of Endocrinology, Diabetology and Nephrology, Department of Internal Medicine, University of Tuebingen, Tuebingen, Baden-Wuerttemberg, Germany; 2 Institute for Diabetes Research and Metabolic Diseases (IDM), Helmholtz Center Munich, University of Tuebingen, Tuebingen, Baden-Wuerttemberg, Germany; 3 German Center for Diabetes Research (DZD), Munich-Neuherberg, Germany; 4 Department for Diagnostic Laboratory Medicine, Institute for Clinical Chemistry and Pathobiochemistry, University Hospital Tuebingen, Tuebingen, Baden-Wuerttemberg, Germany; Weill Cornell Medical College Qatar, QATAR

## Abstract

**Aims:**

Insulin sensitivity and insulin secretion can be estimated by multiple indices from fasting blood samples or blood samples obtained during oral glucose tolerance tests. The test-retest reliability of these indices in repeated measurements within the same individuals can strongly vary.

**Methods:**

We analyzed data of persons without diabetes who underwent two repeated OGTTs. For each measurement pair, we calculated multiple commonly used indices for the assessment of insulin secretion and insulin sensitivity. We then evaluated the coefficient of variation (standard deviation/mean) and discriminant ratio for each index.

**Results:**

89 persons underwent two OGTTs with a median interval of 86 days (IQR 64–249). Among indices of insulin sensitivity derived from fasting blood samples, the revised quantitative insulin sensitivity check index had the smallest coefficient of variation (2.8 ± 2.1%) whereas the C-peptide based homeostasis model assessment 2 had the highest discriminant ratio (1.97 (1.65–2.39)). As for insulin sensitivity indices that are based on OGTT, the oral glucose insulin sensitivity index had the smallest coefficient of variation (6.5 ± 5.1%). The highest discriminant ratio was found for the non-esterified fatty acids-based insulin sensitivity index (NEFA-ISI, 2.70 (2.30–3.22)). For the assessment of insulin secretion from fasting variables, the lowest mean coefficient of variation was found for C-peptide based homeostasis model assessment 2 beta with 10.8 ± 8% and the highest discriminant ratio for the C-peptide / Glucose-Ratio (2.18 (1.84–2.63)). Among indices assessing insulin secretion from an OGTT, the lowest coefficient of variation was found for the ratio of the areas under the C-peptide and glucose curves from 0 to 120 minutes with 11.3 ± 9.7%.

**Conclusion:**

The data reveal large differences in the reproducibility and the discrimination capability of different indices that assess insulin sensitivity or insulin secretion. Our findings can aid the selection of an appropriate index in clinical studies.

## Introduction

About 422 million people worldwide have diabetes mellitus with numbers expected to rise. Impaired insulin secretion and insulin resistance are the two key abnormalities which underlie the pathogenesis of type 2 diabetes.

The gold standard measurement of insulin secretion and insulin sensitivity are the hyperglycemic clamp and the hyperinsulinemic euglycemic clamp [1, 2], respectively. However, these methods are relatively invasive, labor intensive, as well as time-consuming and therefore not feasible in larger clinical studies. Therefore, different approaches to substitute the clamp technique for the assessment of insulin secretion or insulin sensitivity have been proposed. These methods estimate insulin secretion or insulin sensitivity from variables related to these modalities such as insulin, C-peptide, glucose or non-esterified fatty acid (NEFA) levels. NEFAs are tightly linked to insulin sensitivity due to insulin’s role in the suppression of lipolysis [3–5]. Some of the estimates utilize only measurements from fasting blood samples while others employ variables obtained during standardized metabolic challenges such as the oral glucose tolerance test (OGTT). Based on the type of estimate and underlying blood specimen, the measures can capture different aspects of insulin sensitivity or insulin secretion. The OGTT is widely used in clinical practice and derived measures quantify the efficiency of glucose utilization under standard conditions including both hepatic as well as muscle insulin sensitivity [6, 7]. In contrast, insulin sensitivity indices that are solely based on fasting parameters reflect mostly hepatic insulin sensitivity [8]. Most indices of insulin sensitivity and some for insulin secretion have already been evaluated for correlation with gold standard measurements [9, 10].

Certainly, there is an inherent biological and analytical variability in every measurement [11, 12]. In addition to the desired feature to be measured, glycemic traits are highly influenced by other confounding factors such as quality of sleep, subclinical conditions or inflammation [13, 14]. Glycemic variables obtained during an OGTT are further impacted by the speed of gastric emptying [15], rate of incretin release [16] and potential interactions with the gut microbiome. Therefore, test-retest reliability, i.e. the intra-individual concordance of subsequent measurements, can strongly vary among these indices. Though, there is still lack of scientific data regarding this variability. Our work therefore aims to compare the reproducibility for different frequently reported indices for insulin sensitivity and insulin secretion using highly standardized repeated measurements within the same subjects.

## Methods

### Subjects

We analyzed data from 120 persons who underwent two or more oral glucose tolerance tests (OGTT) with a time difference of 4 to 363 days. Subjects analyzed in the current study are participants of a number of clinical studies. These include the Tuebingen Family Study (TUEF, n = 15) [17], Tuebinger-Lebensstil-Interventions-Programm (TULIP, n = 4), Prädiabetes Lebensstil Interventions-Studie (PLIS, NCT01947595, n = 2), Deutsche Studie Schwangerschaftsdiabetes (PREG, n = 16, NCT04270578) as well as 14 other projects (NCT02991365 (n = 1), NCT03151590 (n = 7), NCT03231839 [18] (n = 1), NCT 04372849 (n = 2), NCT01635114 [19] (n = 22), NCT03615209 (n = 15), NCT 03590561 (n = 3), and DRKS00012996 (n = 1)). The research took place in Tuebingen, Baden-Wurttemberg, Germany, between August 2002 and March 2019. The first OGTT was performed between 08/2002 and 02/2019, the second between 05/2003 and 03/2019. Written informed consent was obtained from all participants. A vote of approval for all studies has been obtained from the ethics committee of the University Hospital of Tuebingen.

Persons who had diabetes mellitus defined as either an HbA1c ≥6.5%, fasting glucose ≥7mmol/l or a post-challenge glucose ≥11.1mmol/l at 120 minutes after glucose challenge, who underwent a lifestyle modification or who were pregnant were previously excluded. Furthermore, persons who showed a difference of three or more kilograms body weight between the two glucose tolerance tests (n = 31) were excluded. For persons who underwent more than two oral glucose tolerance tests, the two tests with the lowest time interval were analyzed. Data of 89 persons in accordance with 178 OGTTs remained.

### Oral glucose tolerance test and laboratory measurements

All participants underwent an OGTT with a standardized 75 g glucose solution (Accu-Check Dextro, Roche) after overnight fasting. Venous plasma and serum samples were obtained before ingestion (at minute 0) and 30, 60, 90 and 120 minutes after the glucose challenge. All blood samples were immediately put on ice and the serum was centrifuged within two hours. Plasma glucose was determined in an ADVIA 1800 autoanalyzer (Siemens Healthcare Diagnostics, Erlangen, Germany). Plasma insulin and C-peptide were measured by an immunoassay with the ADVIA Centaur XP Immunoassay System (Siemens Healthineers, Eschborn, Germany). NEFA concentrations were measured enzymatically (WAKO Chemicals, Neuss, Germany) using the ADVIA 1800 analyzer (Siemens Healthcare Diagnostics, Eschborn, Germany). C-reactive protein levels were measured via immunoturbidimetry with the wide range reagents using the ADVIA 1200 analyzer (Siemens, Eschborn, Germany).

### Indices

We used the following common indices for insulin sensitivity and insulin secretion for our study. The indices used are without any claim to completeness.

*Calculated indices to measure insulin sensitivity using parameters from fasting state are*:

- Quantitative insulin sensitivity check index [20] (QUICKI):
$$QUICKI=\frac{1}{log(I_{0})+log(G_{0})}$$
- Revised quantitative insulin sensitivity check index [21] (revised QUICKI):
$$revised\;QUICKI=\frac{1}{log(G_{0})+log(I_{0})+logNEFA}$$
- C-peptide based homeostasis model assessment 2 sensitivity [22] (HOMA2-S_Insulin_):
                           see HOMA2Calculator [23]
- C-peptide based homeostasis model assessment 2 sensitivity [22] (HOMA2-S_C-Peptide_):
                           see HOMA2Calculator [23]
  *Calculated indices to measure insulin sensitivity using parameters from oral glucose tolerance tests are*:
- oral glucose insulin sensitivity index [24] (OGIS):
$$OGIS=f(G_{0},G_{90},G_{120},I_{0},I_{90},D_{0})$$
  whereby *f* is a complex function (which can be programmed on a spreadsheet [25])
- Belfiore index [26] (Belfiore):
$$Belfiore=\frac{2}{(AUC_{Insulin}*AUC_{Glucose})+1}$$
- Non-esterified fatty acids insulin sensitivity index [27] (NEFA-ISI):
$$NEFA-ISI_{0,60,120}=60*e^{3.853-0.9*\ln BMI-0.205*\ln insulin_{0}-0.128*\ln insulin_{60}-0.256*lninsulin_{120}-0.138*lnNEFA_{120}}$$
- Gutt insulin sensitivity index [28] (Gutt):
$$Gutt=\frac{7500+(G_{0}-G_{120})*0.19*bodyweight}{120*G_{mean}*log(I_{mean})}$$
- Matsuda insulin sensitivity index [7] (Matsuda-ISI):
$$Matsuda-ISI=\frac{10000}{\sqrt{\left(G_{0}*I_{0})*(G_{mean}*I_{mean}\right)}}$$
- Stumvoll insulin sensitivity index [29] (Stumvoll-ISI):
$$Stumvoll-ISI=0.226-0.0032*BMI-0.0000645*I_{120}-0.00375*G_{90}$$
- Stumvoll metabolic clearance rate [29] (Stumvoll-MCR):
$$Stumvoll-MCR=18.8-0.271*BMI-0.0052*I_{120}-0.27*G_{90}$$

*Calculated indices to measure insulin secretion using parameters from fasting state are*:

- C-peptide based homeostasis model assessment 2 beta [22] (HOMA2-B_C-Peptide_):
                           see HOMA2Calculator [23]
- insulin based homeostasis model assessment 2 beta [22] (HOMA2-B_Insulin_):
                           see HOMA2Calculator [23]
- C-Peptide/Glucose-Ratio (CGR):
$$CGR=\frac{C_{0}}{G_{0}}$$
  whereby blood glucose levels are used in mg/dl

*Calculated indices to measure insulin secretion using parameters from oral glucose tolerance tests are*:

- Area under the curve (calculated by using the trapezoidal rule) of C-peptide from 0 to 30 minutes (AUC_C-Peptide0-30_)
- Area under the curve of C-peptide from 0 to 120 minutes (AUC_C-Peptide0-120_)
- Ratio of the area under the curve for insulin from 0 to 30 minutes to the area under the curve for glucose from 0 to 30 minutes (AUC_Insulin0-30_/AUC_Glucose0-30_)
- Ratio of the area under the curve of insulin from 0 to 120 minutes to the area under the curve of glucose from 0 to 120 minutes (AUC_Insulin0-120_/AUC_Glucose0-120_)
- Ratio of the area under the curve of C-peptide from 0 to 30 minutes to the area under the curve of glucose from 0 to 30 minutes (AUC_C-Peptide0-30_/AUC_Glucose0-30_)
- Ratio of the area under the curve of C-peptide from 0 to 120 minutes to the area under the curve of glucose from 0 to 120 minutes (AUC_C-Peptide0-120_/AUC_Glucose0-120_)
- Insulinogenic index [30] (IGI):
$$IGI=\frac{\Delta I_{0,30}}{\Delta G_{0,30}}$$
- Oral disposition index [31] (DI):
$$DI=\frac{\Delta I_{0,30}}{\Delta G_{0,30}}*\frac{1}{I_{0}}$$

It is important to emphasize that indices based on insulin concentration should be differentiated from indices based on C-peptide concentration. Whereas indices based on insulin concentration reflect a combination of insulin secretion and insulin clearance and therefore can be seen as a surrogate index of the “body insulin response” to glucose, indices based on C-peptide reflect insulin secretion only and can therefore be seen as an index of “beta cell function”.

The different indices with the respective necessary measurements are summarized in *Table 1.*

**Table 1:** Calculation of the analyzed indices.

		Index	timepoints	variables needed	equation
**Insulin secretion**	**fasting**	HOMA2-B C-peptide	0	Glucose, C-peptide	https://www.dtu.ox.ac.uk/homacalculator/index.php
		HOMA2-B Insulin	0	Glucose, Insulin	https://www.dtu.ox.ac.uk/homacalculator/index.php
		CGR	0	Glucose, C-Peptide	$\frac{C_{o}}{G_{o}}$
	**OGTT**	AUC C-peptide 0-120/AUC Glucose 0–120	0, 120	Glucose, C-peptide	AUC_C-Peptide0-120_/AUC_Glucose0-120_
		AUC C-peptide 0–120	0, 120	C-peptide	AUC_C-Peptid0-120_
		AUC C-peptide 0-30/AUC Glucose 0–30	0, 30	Glucose, C-peptide	AUC_C-Peptide0-30_/AUC_Glucose0-30_
		AUC C-peptide 0–30	0, 30	C-peptide	AUC_C-Peptide0-30_
		AUC Insulin 0-120/AUC Glucose 0–120	0, 30	Glucose, Insulin	AUC_Insulin0-120_/AUC_Glucose0-120_
		AUC Insulin 0-30/AUC Glucose 0–30	0, 30	Glucose, Insulin	AUC_Insulin0-30_/AUC_Glucose0-30_
		IGI	0, 30	Glucose, Insulin	$\frac{{\Delta I}_{0,30}}{{\Delta G}_{0,30}}$
		DI	0, 30	Glucose, Insulin	$\frac{{\Delta I}_{0,30}}{{\Delta G}_{0,30}}*\frac{1}{I_{0}}$
**Insulin sensitivity**	**fasting**	revised QUICKI	0	Glucose, Insulin, NEFA	$\frac{1}{\log\left(G_{0}\right)+\log\left(I_{0}\right)+logNEFA}$
		QUICKI	0	Glucose, Insulin	$\frac{1}{\log\left(I_{0}\right)+log(G_{0})}$
		HOMA2-S C-peptide	0	Glucose, C-peptide	https://www.dtu.ox.ac.uk/homacalculator/index.php
		HOMA2-S Insulin	0	Glucose, Insulin	https://www.dtu.ox.ac.uk/homacalculator/index.php
	**OGTT**	OGIS	0, 90, 120	Glucose, Insulin, height, body weight, glucose dose [g]	$f(G_{0},G_{90},G_{120},I_{0},I_{90},D_{0})$ whereby *f* is a complex function which can be programmed on a spreadsheet[30]	whereby *f* is a complex function which can be programmed on a spreadsheet[30]
		Belfiore	0(, 60), 120	Glucose, Insulin	$\frac{2}{({AUC}_{Insulin}*{AUC}_{Glucose})+1}$
		NEFA-ISI	0, 60, 120	Insulin, NEFA, BMI	$60*e^{\begin{array}{c} (3.853-0.9*lnBMI-0.205*ln{Insulin}_{0}\\ -0.128*ln{Insulin}_{60}-0.256*ln{Insulin}_{120}\\ -0.138*ln{NEFA}_{120}) \end{array}}$ $\frac{7500+(G_{0}-G_{120})*0.19*bodyweight}{120*G_{mean}*log(I_{mean})}$	
		Gutt	0, 120	Glucose, Insulin, body weight	
		Matsuda-ISI	0, 30, 60, 90, 120	Glucose, Insulin	$\frac{10000}{\sqrt{\left(G_{0}*I_{0})*(G_{mean}*I_{mean}\right)}}$

### Statistics

To investigate if there is an association between the coefficient of variation of an index and the time-span between measurements within the prespecified one-year time interval, we fitted linear regression models (CoV_Index_ ~ Δ_time (days)_) for all investigated indices using a multiple-testing corrected α = 0.0024 threshold (see *S1 Table*).

We computed the coefficient of variation (standard deviation/ mean) for each measurement pair to determine the reliability of repeated measurements.

The coefficient of variation is a well-established method for assessing the imprecision of a test. To also account for the capability of a given test to discriminate different subjects in relation to the robustness of within subject measurements, we also calculated the discriminant ratio (DR) as proposed Levy et. al [32] as follows:

$$DR=\sqrt{\frac{{MS}_{B}-{MS}_{W}}{k∙{MS}_{W}}}$$

Where MS_B_ and MS_W_ are the between-subject and within subject mean squares respectively and k is the number of repeated measurements within subject. The discriminant ratio is higher when the differences between subjects are higher and within-subjects are lower. Confidence intervals for discriminant ratios were computed using non-central F distributions and differences between the discriminant ratios in the index groups were tested using Q-statistic, as recommended [32].

All computations were performed with R version 3.6.3 [33].

### Literature research

To assess how often different indices are used, we researched the literature via the ISI web of knowledge using the search function title. As key words we used the full title of each original article. The original articles are cited in the upper part (see section Methods-Indices). We looked for the number of citations of the original articles of each investigated index, where possible. All studies found were included. The literature research was conducted on 26th of February 2020. Unfortunately, it was not possible to differentiate various HOMA2 indices in our literature research. Due to difficulties in identifying indices containing areas under curve for C-peptide, insulin and glucose in the literature, it was not possible to assess the citation number for these indices.

## Results

We analyzed data of 178 OGTTs from 89 persons (51 females, 38 males). For each of them, data from two OGTTs with a median of 86 days (IQR 64–249) between the two measurements were analyzed. Participants had a median age of 42 years (IQR 29–57) and a median body mass index (BMI) of 27.9 kg/m^2^ (IQR 23.4–32.4). An overview of the participants`characteristics is given in *Table 2*.

**Table 2:** Characteristics of participants.

characteristic	
**Total number of participants**	89
**Sex [n]**	
male	38
female	51
**Age [years]**	
median (interquartile range)	42 (29–57)
range	18–72
**BMI [kg/m** ^**2**^ **]**	** **
median (interquartile range)	27.9 (23.4–32.4)
range	17.7–42.0
**time difference [days]**	
median (interquartile range)	86 (64–249)
range	4–363

In *Table 3* the mean and the standard deviation of the coefficient of variation as well as the discriminant ratio for each calculated index arranged by insulin secretion or insulin sensitivity as well as by the sampling method (fasting or OGTT) are shown. Moreover, the mean per group is shown here.

**Table 3:** Coefficient of variation and discriminant ratio index-wise and per group.

		**Index**	CoV mean^1^	CoV sd^2^	DR (95% CI)^3^
**Insulin secretion**	**fasting**	HOMA2-B C-peptide	0.108	0.080	1.69 (1.39–2.07)
		CGR	0.134	0.101	2.18 (1.84–2.63)
		HOMA2-B Insulin	0.142	0.116	1.58 (1.28–1.96)
		**group^4^**	0.128	0.099	1.82 (1.50–2.22)
	**OGTT**	AUC C-peptide 0-120/AUC Glucose 0–120	0.113	0.097	2.13 (1.79–2.57)
		AUC C-peptide 0–120	0.127	0.095	2.28 (1.93–2.74)
		AUC C-peptide 0-30/AUC Glucose 0–30	0.155	0.157	1.71 (1.41–2.09)
		AUC C-peptide 0–30	0.160	0.157	1.79 (1.48–2.18)
		AUC Insulin 0-120/AUC Glucose 0–120	0.161	0.112	2.52 (2.14–3.01)
		AUC Insulin 0-30/AUC Glucose 0–30	0.198	0.158	1.76 (1.45–2.14)
		IGI	0.273	0.233	0.57 (0.23–0.86)
		DI	0.288	0.252	0.12 (0–0.53)
		**group**	0.184	0.158	2.03 (1.30–2.02)
**Insulin sensitivity**	**fasting**	revised QUICKI	0.028	0.021	1.97 (1.65–2.39)
		QUICKI	0.044	0.036	1.94 (1.62–2.34)
		HOMA2-S C-peptide	0.149	0.117	1.97 (1.65–2.39)
		HOMA2-S Insulin	0.210	0.173	1.40 (1.12–1.75)
		**group**	0.108	0.087	1.82 (1.51–2.22)
	**OGTT**	OGIS	0.065	0.051	1.99 (1.67–2.40)
		Belfiore	0.112	0.096	1.52 (1.23–1.87)
		NEFA-ISI	0.128	0.085	2.70 (2.30–3.22)
		Gutt	0.173	0.140	1.24 (0.97–1.56)
		Matsuda-ISI	0.188	0.150	2.16 (1.82–2.59)
		**group**	0.133	0.104	1.92 (1.59–2.33)

^1^ mean coefficient of variation.

^2^ standard deviation of the coefficient of variation.

^3^ discriminant ratio with the corresponding 95% confidence interval.

^4^ representing the mean values for each group (insulin secretion fasting, insulin secretion OGTT, insulin sensitivity fasting, insulin sensitivity OGTT).

For the indices derived from **fasting** blood that assess **insulin sensitivity**, the smallest mean coefficient of variation was found for revised QUICKI at 2.8% (± 2.1%). With 4.4% (± 3.6%) QUICKI also showed a low coefficient of variation while HOMA2-S_C-Peptide_ with 14.9% (± 11.7%) and HOMA2-S_Insulin_ with 21.0% (± 17.3%) demonstrate larger coefficients of variation.

There were no differences among discriminant ratios in the group of fasting-based insulin sensitivity indices (p = 0.07, see Fig 2C).

For indices that use measurements from **oral glucose tolerance tests** to measure **insulin sensitivity**, the smallest coefficient of variation was found for OGIS with 6.5% (± 5.1%) followed by Belfiore with 11.2% (± 9.6%), NEFA-ISI with a mean coefficient of variation of 12.8% (± 8.5%) and Gutt with 17.3% (± 14.0%). With a coefficient of variation of 18.8% (± 15.0%), the Matsuda-ISI had the highest figure in this group.

The highest discriminant ratio was found for NEFA-ISI (2.70 (2.30–3.22) followed by Matsuda-ISI (2.16 (1.82–2.59) and OGIS (1.99 (1.67–2.40)). With 1.52 (1.23–1.87) Belfiore showed a lower discriminant ratio followed by Gutt with 1.24 (0.97–1.56).

For the indices for **insulin secretion** that use **fasting variables**, the lowest coefficient of variation was found for HOMA2-B_C-Peptide_ with 10.8% (± 8%) followed by CGR with 13.4 (± 10.1%) and HOMA2-B_Insulin_ with 14.2% (± 11.6%) whereas the highest discriminant ratio was found for CGR with 2.18 (1.84–2.63).

Comparing the indices using **OGTT-based variables** to assess **insulin secretion**, the smallest coefficient of variation at 11.3% (± 9.7%) was found for AUC_C-Peptide0-120_/AUC_Glucose0-120_. The different coefficients of variation of AUCs are shown in *Table 3*. The largest coefficient of variation for measuring insulin secretion from OGTT-derived variables was demonstrated for IGI with 27.3% (± 23.3%) and DI with 28.8% (± 25.2%).

We have also performed a sensitivity analysis of the coefficients of variation after excluding female premenopausal participants (see *S2* and *S3* Tables).

With regard to the discriminant ratio, the highest value was found for AUC_Insulin 0-120_/AUC_Glucose0-120_ with 2.52 (2.14–3.01) and the lowest for DI (0.12 (0–0.53). Taking both, the coefficient of variation and the discriminant ratio into account, CGR and HOMA2-B_C-Peptide_ seem to be superior to HOMA2-B_Insulin_ in the group assessing insulin secretion using fasting values. Furthermore, AUC_C-Peptide0-120_/AUC_Glucose0-120_, AUC_C-Peptide0-120_ and AUC_Insulin0-120_/AUC_Glucose0-120_ seem to be superior to the other OGTT-based tests assessing insulin secretion.

For indices assessing insulin sensitivity using fasting variables, HOMA2-S_Insulin_ shows the highest coefficient of variation and the lowest discriminant ratio. The best performing index for discrimination between different subjects in relation to its variability is the NEFA-ISI with a low coefficient of variation.

**Fig 1:**
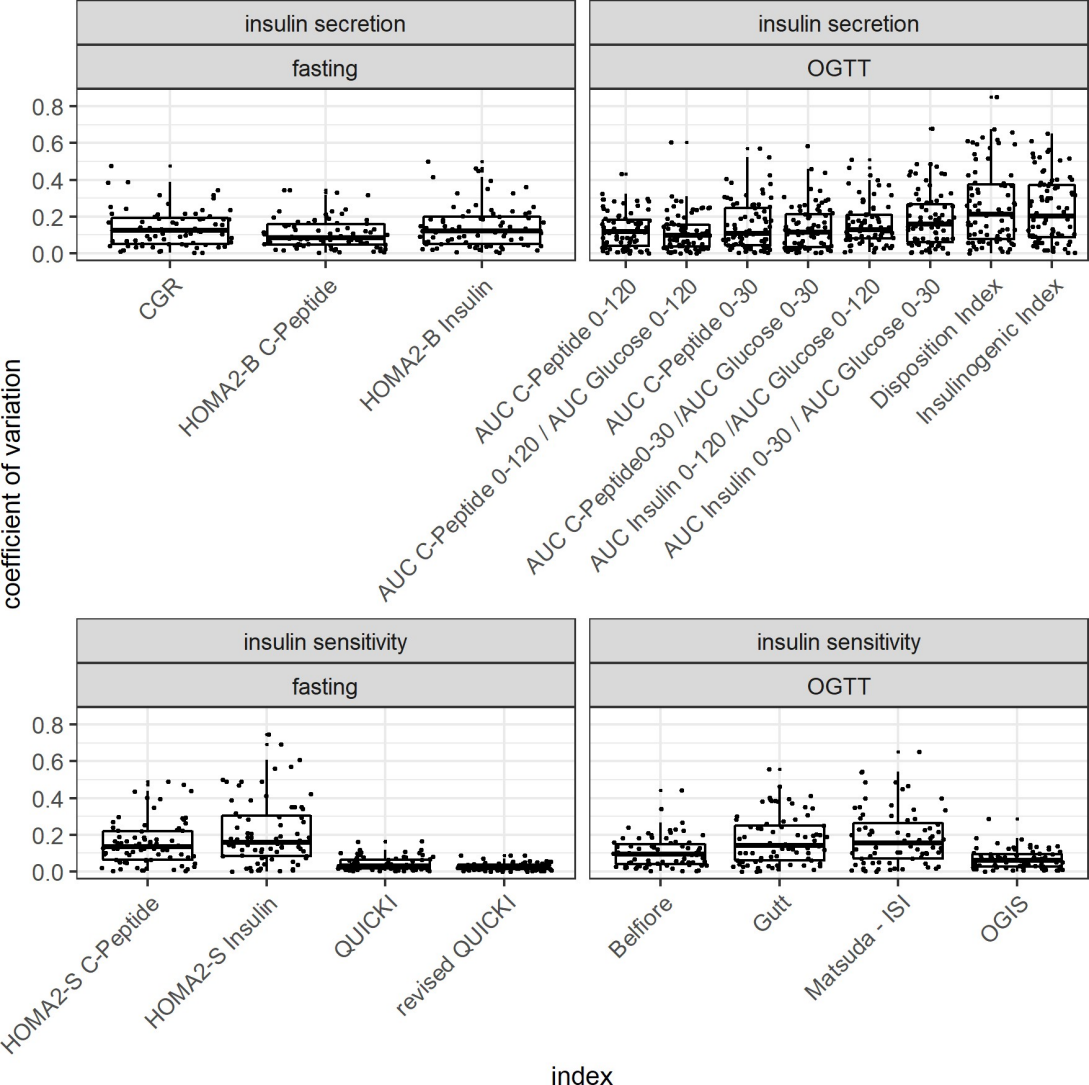
Boxplot showing coefficients of variation of the analyzed indices of insulin secretion and insulin sensitivity.

**Fig 2:**
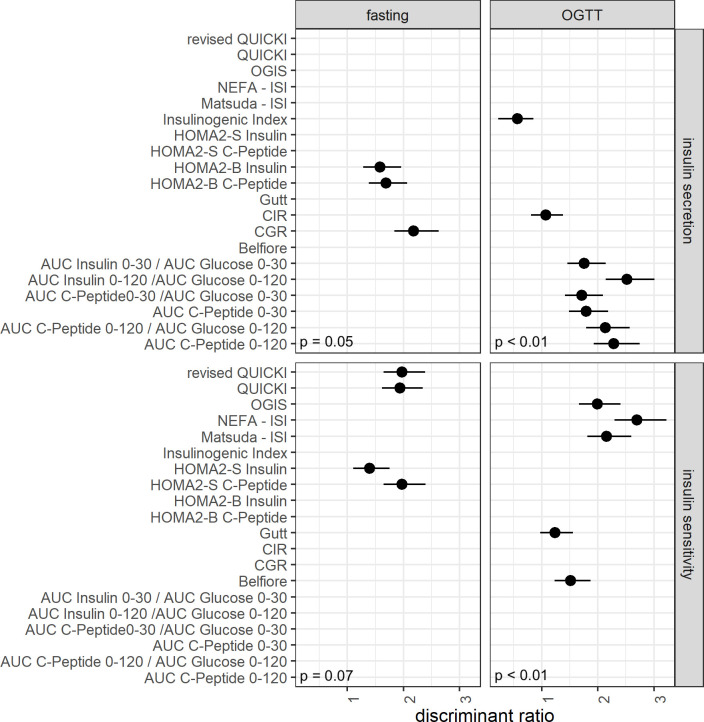
Discriminant ratio and corresponding confidence intervals of the analyzed indices of insulin secretion and insulin sensitivity. The disposition index had been omitted due to a very low point estimate (0.12) and a failure to compute the confidence interval. The discriminant ratios and their confidence intervals, as well as the p-values of the tests of differences within groups were computed as proposed by Levy at al [32].

*Fig 1* shows a boxplot of the coefficients of variation of the different indices arranged by the fore mentioned order. *Fig 2* shows the discriminant ratios and the corresponding confidence interval for each index.

A groupwise aggregation of different coefficients of variation (showed a mean of 12.8% (± 9.9%) in the insulin secretion from fasting measures group, a mean of 18.4 ± 15.8% in the insulin secretion from OGTT measures group, a mean of 10.8 ± 8.7% in the insulin sensitivity fasting group and a mean of 13.3% (± 10.4%) in the insulin sensitivity OGTT group.

An overview of the mean discriminant ratios for each group shows a mean discriminant ratio of 1.82 (1.50–2.22) for the insulin secretion fasting group and with 2.03 (1.30–2.02) a higher discriminant ratio for the insulin secretion OGTT group. In the insulin sensitivity OGTT group we found a discriminant ratio of 1.92 (1.59–2.33) and a lower discriminant ratio in the insulin sensitivity fasting group with 1.82 (1.51–2.22).

Our literature research yielded inter alia 3415 citations of the original paper of Matsuda, 2452 citations of the original paper of QUICKI, 1198 of the original paper of HOMA2 and 1103 citations of the original paper of DI. The original paper of revised QUICKI was cited 168 times, that of NEFA-ISI 11 times. An overview of the number of citations per year (calculated by the total number of citations divided by the years since first publication) in relation to the coefficients of variation for each group is given in S1 Fig.

## Discussion

With a coefficient of variation range between 2.8 to 28.8%, our data show large differences in the reproducibility of different indices measuring insulin sensitivity and insulin secretion. The intra-individual coefficient of variation of indices using fasting variables is smaller than that of indices using variables measured during an OGTT for both insulin sensitivity and insulin secretion (see *Table 3*). Schousboe et al. already described a larger coefficient of variation in indices measuring insulin sensitivity and secretion than in 2h post-load glucose and in fasting blood glucose [34]. The difference may be due to the greater number of measurements for the OGTT based variables generating more variance and due to variable passage and resorption of the glucose solution after ingestion [34, 35]. Change of insulin sensitivity through the menstrual cycle is controversially discussed as a potential further source of intraindividual variation [36, 37]. To address this, we excluded female premenopausal participants in an additional analysis (see *S2 and S3 Tables*).

The results were comparable to the whole group.

In addition to coefficient of variation, we also computed the discriminant ratio of all indices. The discriminant ratio as recommended by Levy et. Al [32] is an excellent instrument which not only incorporates the imprecision of a test but quantifies this in relation to the capability to distinguish between different subjects.

### Insulin sensitivity

Revised QUICKI [21] and the original QUICKI [20] showed the smallest coefficients of variation among the indices estimating **insulin sensitivity** from **fasting variables** as well as compared to all evaluated indices. Of note, revised QUICKI has been shown to have the highest correlation with gold standard measurements in a meta-analysis [9]. In addition to fasting glucose and insulin, revised QUICKI uses the fasting level of NEFA. NEFA are not measured routinely in most metabolic studies and require precise pre-analytics in order to avoid in vitro lipolysis [38].

Generally, insulin concentrations in the bloodstream have a large biological variability due to the hormones short serum half-life, its pulsatile secretion [39], a marked first-pass effect before reaching systemic circulation. In addition to the biological variance, there is a considerable analytical variability of laboratory insulin measurement approaches [10]. Of note, HOMA2-S_Insulin_ [22] had higher coefficients of variation than QUICKI, despite calculating insulin sensitivity from the same variables.

The lowest coefficient of variation to measure **insulin sensitivity using dynamic parameters** was found for OGIS [24]. OGIS has shown good agreement with the hyperinsulinemic euglycemic clamp and a better correlation in comparison to other indices [24, 40]. Another advantage is that blood sampling is only required at three different time-points during the OGTT (minutes 0, 90 and 120). However, a limitation of OGIS is the complex function, that can, however, be circumvented by web-based calculators and available Excel-sheets [25].

Both the Belfiore [26] and the NEFA-ISI [27] use NEFA levels for the assessment of insulin sensitivity. They exhibit somewhat larger coefficients of variation than OGIS. NEFA-ISI only comprises insulin and NEFA levels at different time-points and it is the only insulin sensitivity index that does not utilize glucose concentrations at all. This makes it more accurate to measure insulin sensitivity in special situations like pregnancy, a state with physiologically lower glucose levels that often hinders comparison of pregnant with non-pregnant women.

The Matsuda index [7] correlates well with insulin sensitivity assessed from euglycemic hyperinsulinemic clamp [41]. It is calculated from fasting glucose and insulin levels and the mean of glucose and insulin during OGTT [7]. Matsuda, computed from 5-point OGTT data, has shown an intraindividual coefficient of variation of 18.8% which is the largest in the group of indices measuring insulin sensitivity with OGTT parameters. The higher number of measured points may explain the higher coefficient of variation as well as the fore mentioned higher variability of insulin and glucose levels.

When comparing the computed coefficients of variations and the discriminant ratios between groups, fasting-based indices showed lower coefficients of variation (10.8%) but also lower discriminant ratios (1.82) than OGTT-based indices (coefficient of variation 13.3% and discriminant ratio 1.92). This might indicate that the indices using fasting values might be more stable during repetitive measurements, but have less power to discriminate metabolic differences between subjects.

Stumvoll-ISI and Stumvoll-MCR incorporate demographic data as age, sex and BMI in addition to glucose and insulin levels obtained during an OGTT. These are reliable indices [29]. Unfortunately, in our group of subjects we found several negative indices in persons with high insulin levels 120 minutes after glucose load so that we excluded these indices from these analyses.

### Insulin secretion

The lowest coefficient of variation in the measurement of **insulin secretion from fasting parameters** was shown for HOMA2-B_C-Peptide_. This is most likely due to the above mentioned difficulties in insulin measurements. C-peptide and insulin are secreted in equimolar amounts based on their cleavage out of proinsulin. It is a more stable parameter than insulin and has insignificant clearance by the liver and a longer half-life than insulin [42]. Therefore C-peptide levels better approximate pancreatic insulin secretion than insulin levels [43], and so the use of C-peptide to compute HOMA2-B has clear advantages. In clinical practice, it has been recently suggested that a simple ratio of plasma C-peptide over plasma glucose in mg/dl could result in a straight fore ward classification of insulin secretion capacity. Our data show that this ratio has a comparable coefficient of variation to the other indices of this category.

The OGTT had been described as an acceptable compromise to assess ß-cell function [44]. When estimating **insulin secretion from dynamic parameters during an oral glucose challenge** the lowest coefficient of variation was found for AUC_C-Peptide0-120_/AUC_Glucose0-120_ (11.3% ± 9.7%). In comparison, the coefficient of variation of AUC_Insulin0-120_/AUC_Glucose0-120_ was larger (16.1% ± 11.2). This is well in line with the noted superiority of C-peptide over direct insulin measurements. In this category of insulin secretion from dynamic parameters during an oral glucose challenge the highest coefficient of variation was found for DI. This index has advantages as it is an early marker of an inadequate compensation of beta cell function [45]. The assumption on which the measurement was established is that all measurements of insulin sensitivity or response show a hyperbolic relationship. But there are discussions if this assumption is sufficient [31]. Besides, the variability of insulin levels may be a source of high variability as well.

As for the group of indices measuring insulin sensitivity, our study shows the same differences when comparing the computed coefficient of variation and the discriminant ratios in the insulin secretion groups. The group based on fasting values shows a smaller coefficient of variation (12.8%) and a smaller discriminant ratio (1.82) than the OGTT group (coefficient of variation 15.8% and discriminant ratio 2.03)

Utzschneider et al. already described a high within-subject variability of various indices and proposed integrated measurements using multiple time points and C-peptide levels to reduce variability [46]. The study population Utzschneider et al. describe consists of overall 37 persons of whom some had normal glucose tolerance, some had impaired glucose tolerance and some had diabetes–with or without medication. Our study conversely just analyzed people without diabetes to ensure a better comparability. Moreover, our study population was larger so that the generalisability should be better. Despite the differences in the sample size and the study population, all in all, our data is in line with the findings of Utzschneider`s study with a high coefficient of variation for insulinogenic index and AUCs with smaller coefficients of variation. One could therefore assume that the variability of the different indices is independent of the glucose levels. This should be investigated by other studies.

Studies comparing the coefficient of variation and the discriminant ratio at the same time are rare. The only study which we are aware of is by Mather et al. [10]. Mather et al. measured the repeatability of different tests of insulin sensitivity. In their investigation logHOMA-IR and QUICKI provided the best results with a low coefficient of variation and a high discriminant ratio.

Similar results were found in our study. QUICKI and also revised QUICKI showed a low coefficient of variation concomitant with a high discriminant ratio. Additionally, we investigated more indices and also the insulin secretion. In the insulin sensitivity group OGIS and NEFA-ISI are two important indices which seem to be superior to the other OGTT-based insulin sensitivity indices.

The discussed indices are commonly used indices. However we did not cover one group of indices in our work, the indices computed by mathematical modeling. For example Mari et al. established models for beta-cell assessment [44, 47]. Most indices based on mathematical modelling require a more frequent glucose sampling, especially a measurement point at 15 minutes.

### Use of indices in the literature

QUICKI is used very often, most likely as it only requires one fasting blood sampling. Our literature research showed 2,452 citations, i.e. the second highest number of citations of all used indices. Conversely, the original paper of revised QUICKI was cited just 168 times. Although OGIS has shown good agreement with the hyperinsulinemic euglycemic clamp and we now verified a favorable test-retest coefficient of variation, it is not used applied very frequently. This might be partly due to its complexity, despite the available web-based calculators and Excel-sheets [25]. With 514 citations of the original paper in our literature research it is less cited than the Matsuda index. Matsuda index with 3415 citations is the most cited paper in this category and among all evaluated indices. Of note, this top-cited index shows the highest coefficient of variation.

### Limitations

Our study investigated several indices for reliability during repeated measurements. Due to the single-center design, our highly standardized study settings and laboratory measurements, we potentially reduced variance that can originate from methodological and pre-analytic and analytical differences in routine clinical use. Our work only addressed the coefficient of variation and discrimination ratios of the indices and did not compare how these indices relate to gold-standard measurements. Furthermore we did not cover the group of secretion/sensitivity indices computed by mathematical modeling. Most indices based on mathematical modeling require a more frequent glucose sampling. Another limitation may lay in the small number of OGTTs which could restrict the generalisability of our data.

## Summary and outlook

Reliable estimation of insulin sensitivity and secretion is crucial when investigating glucose metabolism in clinical research. Furthermore, measurement of these key metabolic features recently gained clinical importance with the introduction of newly identified subphenotypes of prediabetes and diabetes and their potential impact towards more personalized strategies for diabetes therapy. Our current work can aid the selection of the appropriate index in upcoming clinical studies on insulin sensitivity or insulin secretion.

## Supporting information

S1 Fig Coefficients of variation in relation to the number of citations per year. Coefficients of variation of the analyzed indices in relation to the number of citations of the original article per year calculated by the total number of citations divided by the years since first publication. Indices that we could not found or differentiate in our literature research or which are not published yet are presented in red using random values for the y axis. Abbreviations: C = C-peptide; I = Insulin; G = Glucose.
(TIF)

S1 Table Linear regression model between coefficient of variation for each index and time span (days). Samples with a time span more than one year between the two OGTTs are already excluded. Linear regression models (CoV_Index_ ~ Δ_time (days)_) for all investigated indices using a multiple-testing corrected α = 0.0024 threshold.
(DOCX)

S2 Table Coefficient of variation for in postmenopausal female participants and male participants (n = 72) (premenopausal female participants (n = 17) excluded). (DOCX)

S3 Table Characteristics of participants in an analysis of postmenopausal female participants and male participants (n = 72) (premenopausal female participants (n = 17) excluded). (DOCX)
