# Supplementary figures and images for: Reproducibility and discrimination of different indices of insulin sensitivity and insulin secretion
Source: PLoS One. 2021 Oct 22;16(10):e0258476. doi: 10.1371/journal.pone.0258476 (PMC8549015; doi:10.1371/journal.pone.0258476)

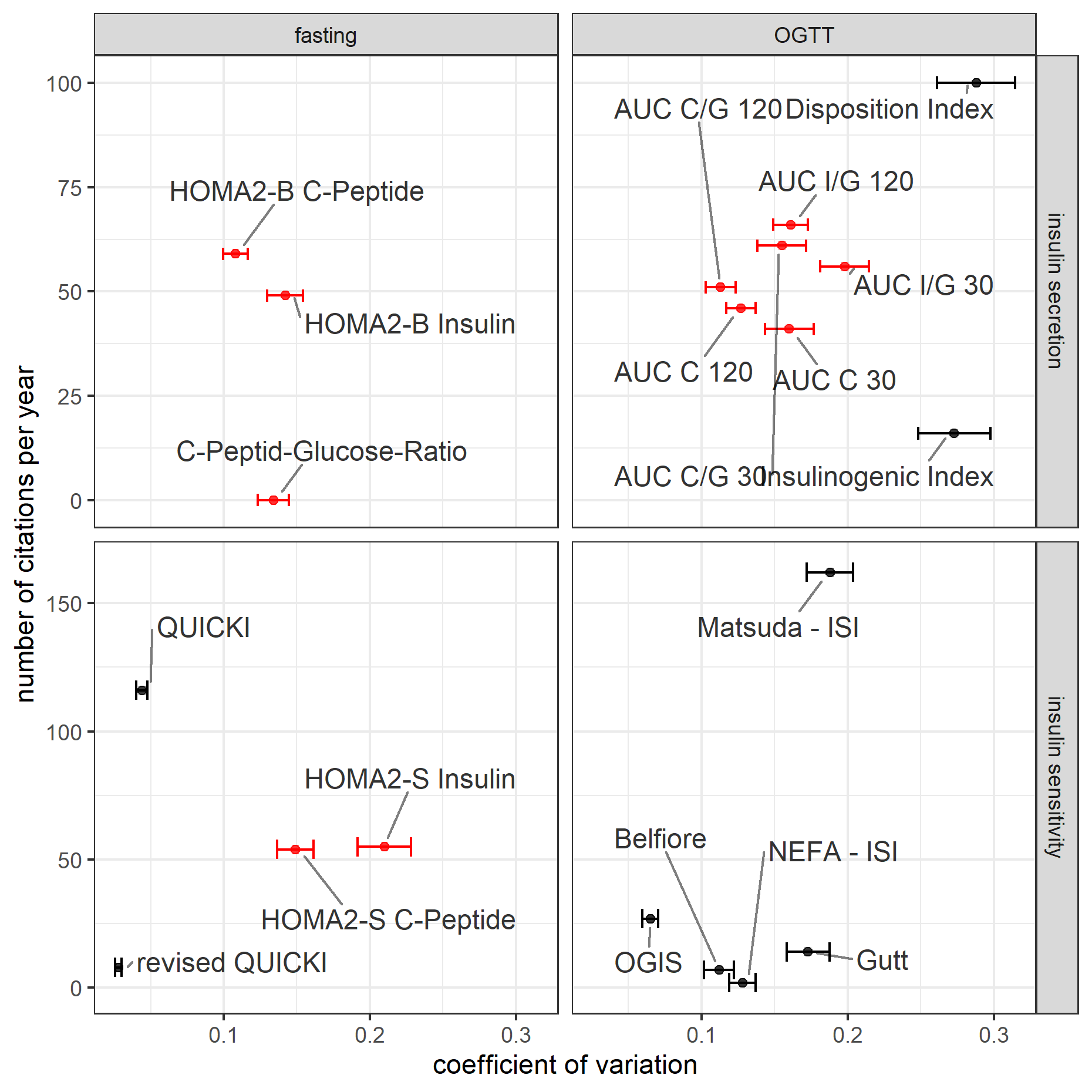

Supplement: S1 Fig — Coefficients of variation of the analyzed indices in relation to the number of citations of the original article per year calculated by the total number of citations divided by the years since first publication. Indices that we could not found or differentiate in our literature research or which are not published yet are presented in red using random values for the y axis. Abbreviations: C = C-peptide; I = Insulin; G = Glucose. (TIF) [file pone.0258476.s001.tif]
