# Supplementary material for: Reproducibility and discrimination of different indices of insulin sensitivity and insulin secretion
Source: PLoS One. 2021 Oct 22;16(10):e0258476. doi: 10.1371/journal.pone.0258476 (PMC8549015; doi:10.1371/journal.pone.0258476)
Supplement: S1 Table — Samples with a time span more than one year between the two OGTTs are already excluded. Linear regression models (CoVIndex ~ Δtime (days)) for all investigated indices using a multiple-testing corrected α = 0.0024 threshold. (DOCX) [file pone.0258476.s002.docx]

**S1 Table. Linear regression model between coefficient of variation for each index and time span (days).**

Table legend:

Samples with a time span more than one year between the two OGTTs are already excluded. Linear regression models (CoV_Index_ ~ ∆_time (days)_) for all investigated indices using a multiple-testing corrected α = 0.0024 threshold.
