# Supplementary material for: Reproducibility and discrimination of different indices of insulin sensitivity and insulin secretion
Source: PLoS One. 2021 Oct 22;16(10):e0258476. doi: 10.1371/journal.pone.0258476 (PMC8549015; doi:10.1371/journal.pone.0258476)
Supplement: S2 Table — (DOCX) [file pone.0258476.s003.docx]

**S2 Table. Coefficient of variation for in postmenopausal female participants and male participants (n=72) (premenopausal female participants (n=17) excluded)**
